# Assessing the Perceptions and Practices Toward Eye Injuries First Aid Among General Population in the Western Region of Saudi Arabia: A Cross-Sectional Study

**DOI:** 10.7759/cureus.36362

**Published:** 2023-03-19

**Authors:** Ammar S Alhothali, Moayad K Aljabri, Osama S Zamzami, Mohammed A Althubaiti, Abdullah S Alshanbari, Abdullah K Alsaeedi, Abdullah Al-Ghamdi

**Affiliations:** 1 Department of Medicine and Surgery, College of Medicine, Umm Al-Qura University, Al-Abdia Main Campus, Makkah, SAU; 2 Department of Medicine and Surgery, Medicine and Surgery, Makkah, SAU; 3 Department of Ophthalmology, Umm-Alqura University, Makkah, SAU

**Keywords:** practice, western region, knowledge, general population, first aid, early management, eye injuries

## Abstract

Aim

The eye is a vital and extraordinarily perceptive part of the human body. A wide spectrum of possible outcomes from eye injuries, from temporary vision loss to permanent blindness. There is a lack of information about how to handle an eye injury. Education and raising awareness are the best way to prevent severe complications and eventual loss of vision. Therefore, the purpose of this study is to evaluate and assess general population knowledge and first aid practices regarding eye injuries in the Western Region of Saudi Arabia, focusing on the importance of early management.

Methodology

A cross-sectional questionnaire was administered among residents of western Saudi Arabia using a validated questionnaire. One thousand two hundred seventy-nine adults of both genders were randomly chosen to represent the sample. In December 2022, we used a web-based survey to gather our data.

Result

This study analyzed data from 1279 responses on the online platform. Results showed that good knowledge was significantly higher among females than males, those without a history of eye injury than their peers with injuries, and those with higher educational levels than those with lower educational levels. Additionally, good knowledge was predicted by the female gender (OR = 1.6, 95% CI, 1.3 to 2.1, p < 0.001).

Conclusion

The study found that public awareness of eye injury first aid was good in the western region. The role of physicians should be expanded and health education campaigns and social media are recommended to achieve the goal of reduction and limiting the most crucial damage to the most sensitive organ in the body.

## Introduction

The eye is one of the most essential senses and delicate organs for every human body since it is one of the most vulnerable organs. Hence, the severity of ocular injuries ranges from relatively minor, non-sight-threatening effects to long-term consequences that may cause blindness [1]. According to the WHO Program for the Prevention of Blindness, there are 55 million eye injuries annually, and approximately 1.6 million become blind due to eye injuries. Additionally, 2.3 million have bilateral impaired vision due to similar issues [2].

Eye injuries present various symptoms, including redness and pain in the injured eye [3]. There are multiple causes such as penetrating globe injuries, foreign corneal substances, corneal abrasions, and even chemical burns. Furthermore, eye injury is the most common reason for ophthalmic emergency department visits [4].

A cross-sectional study conducted in the Asser region of Saudi Arabia measured the awareness and practices of eye injuries and their essential initial management among the general population, revealing poor knowledge [5]. Another study in Riyadh reported that most cases presented to the emergency department were non-emergent and could be managed in the outpatient department [6].

Although there is a shortage of information about dealing with an injured eye, the best way to protect the eyes and vision from severe complications and eventually loss of vision is education and raising awareness of multiple types of eye injury and how to manage and interfere with it appropriately. To the best of our knowledge, there are no articles focused on knowledge and practice about eye injuries, with awareness about the importance of early management, except for the previously mentioned Asser article [5]. Therefore, our study aims to assess the knowledge and practice of eye injury first aid, with awareness about the importance of early management among the general population in the Western Region of Saudi Arabia.

## Materials and methods

Research design and sampling method

A quantitative, observational, cross-sectional study was carried out between December 2022 and February 2023 to assess the knowledge and awareness of the Saudi Arabian population regarding first aid for an eye injury. A non-probability convenience sample of individuals over 18 who speak Arabic for easier communication was included. Visitors to Saudi Arabia were excluded from the study. We used OpenEpi (version 3.0) for sample size calculation: a minimum sample size of 385 was required for the study, considering a 95% confidence interval (CI), an anticipated frequency of 50%, and design effects of one. To improve generalizability and accuracy, we enrolled a total of 1,377 people, which is similar to the sample size of the Asser study (5).

Data collection tools and process

A closed-ended structured questionnaire was adapted from a study in the Asser region of Saudi Arabia [5]. The participants received this questionnaire via social media. Self-administered questionnaires had 23 closed-ended questions, separated into the following categories: demographics (five questions), history, knowledge of eye injury, and its management (18 questions). It consisted of true and false and multiple choice. One thousand two hundred seventy-nine people were involved in this study using an Arabic online questionnaire encompassing Jeddah, Makkah, and Taif. Those who were included in the study were asked to sign a consent form. No participant-identifying information was gathered to ensure confidentiality.

Scoring

In the current study, knowledge items consisted of 17 items. Two things had multiple correct answers (seven correct responses for one item and four correct responses for another item). Therefore, 26 items were relevant to the computation of a knowledge score, calculated by summing up the correct responses. Each correct response was assigned 1, and incorrect answers were assigned zero. A good knowledge level was at >60% of the overall knowledge score.

Statistical analysis

Data analysis was carried out using RStudio (R version 4.1.1). Categorical data were presented as frequencies and percentages. A normality test on the knowledge score revealed non-normally distributed data (p-value for the Shapiro-Wilk normality test < 0.0001). Therefore, the knowledge score was expressed as the median and interquartile range (IQR). A Cronbach alpha test was implemented to assess the internal consistency of knowledge items after assigning correct and incorrect responses. The prevalence of good knowledge was assessed using a one-sample proportion test, and the prevalence was demonstrated along with the respective 95% CIs. Items with multiple correct responses were analyzed using a multiple-response analysis. Factors associated with good knowledge were assessed using a Pearson's Chi-squared test or a Fisher's exact test. Variables with significant associations with participants' knowledge were further used as independent variables in a multivariate binomial logistic regression model to assess the independent predictors of knowledge. The outcomes were presented as odds ratios (ORs) and 95% CIs. A p-value of 0.05 indicated statistical significance.

Ethical considerations

The Umm Al-Qura ethics committee approved this survey for conduct in 2022, ensuring that it was carried out in a way consistent with the values established in Helsinki's Declaration. Participants' names, phone numbers, and identification card numbers were kept confidential. Each respondent provided informed online permission and was informed that the study was voluntary and confidential before taking the survey.

## Results

Sociodemographic characteristics

We received 1,377 responses on the online platform out of a total of 1,500. As a result, the study response rate is approximately 92%. However, we excluded 60 responses from those residing outside the Western region, 30 from those who declined to participate, and eight from those who had missing values. Therefore, data from 1,279 participants were ultimately analyzed (Table 1). More than half of the respondents were females (65.5%), single (54.3%), and had obtained a bachelor's degree (67.6%). In addition, more than a third of the respondents had a monthly income of <5,000 SAR (36.7%).

**Table 1:** Sociodemographic characteristics

Parameter	Category	N (%)
Gender	Male	441 (34.5%)
	Female	838 (65.5%)
Age (y)	18 to 24	564 (44.1%)
	25 to 29	168 (13.1%)
	30 to 34	121 (9.5%)
	35 to 45	223 (17.4%)
	> 45	203 (15.9%)
Marital status	Single	695 (54.3%)
	Married	506 (39.6%)
	Divorced	56 (4.4%)
	Widow	22 (1.7%)
Educational level	< secondary	45 (3.5%)
	Secondary	256 (20.0%)
	University	864 (67.6%)
	Post-graduate	114 (8.9%)
Monthly income (SAR)	< 5,000	470 (36.7%)
	5,000 to 10,000	330 (25.8%)
	10,000 to 15,000	245 (19.2%)
	> 15,000	234 (18.3%)

Description of the knowledge domain

**Figure 1:**
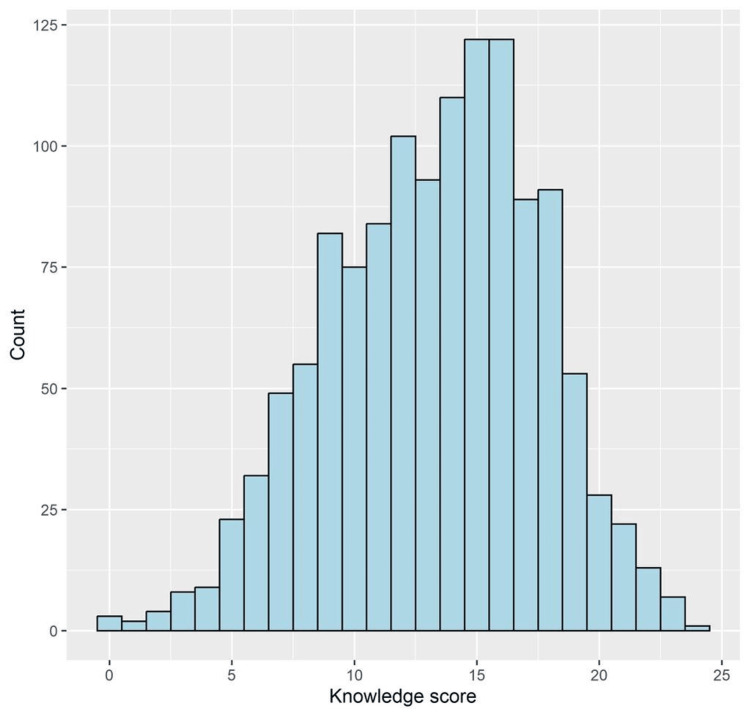
A histogram showing the distribution of the knowledge score.

Reliability analysis showed that the internal consistency of the knowledge items was good (Cronbach alpha = 0.734, number of items = 26). The median (IQR) knowledge score was 14.0 (10.0 to 16.0), with a minimum and maximum of 0 and 24, respectively. The distribution of the knowledge score is depicted in Figure 1. Good knowledge was apparent among 426 participants (33.3%, 95% CI, 30.7 to 36.0).

Responses to knowledge items

The detailed responses to knowledge items are demonstrated in Table 2. It shows that almost one-quarter of them had a history of eye injury (23.8%). In response to the case which requires going to an emergency room, approximately two-thirds of the participants correctly identified persistent eye pain (67.4%), vision problems (66.7%), having torn eyelids (70.0%) and having foreign bodies in the eye (68.3%), whereas 60.8%, 58.4%, and 47.4% have correctly identified having abnormal eye movement in one eye, blood in the eye and changes in the shape of the pupil, respectively.

For a corneal abrasion, the following correct responses were provided for the symptoms: feeling like something stuck in the eye (61.0%), eye pain (60.2%), blurred vision (59.2%), and photophobia (36.7%), while rinsing the eye with saline and clean water was the correct first response to getting a scratched eye by 42.6% of the respondents. Most respondents answered correctly against rubbing their eyes to remove foreign objects (73.7%) and wearing cosmetic contact lenses (87.2%). In contrast, only 30.3% have correctly declined redness-relieving eye drops for a scratched eye.

About two-thirds (66.8%) of the sample correctly stated that a gentle application of a cold compress is the first approach in case of a bruise blow to the eye. In the case of penetrating or a perforating injury, a significant proportion of the participants (78.7%) admitted that a clean protective cover should be applied to the affected eye as a temporary measure before seeing a doctor. However, 47.9% correctly stated that they should not remove anything stuck in the eye, and 16.2% indicated avoiding rinsing with water.

Approximately three-quarters (76.6%) of the respondents agreed that chemical injuries could cause ocular complications. However, washing with plenty of water was correctly selected by 42.1% of the respondents as the first corrective action to chemical injuries. Furthermore, only 25.9% and 20.6% stated that alkaline injuries are more dangerous than acidic ones and that locating and removing particles in the eye was necessary, respectively. Small proportions of the respondents have stated that they should not wash the eye with an alkaline solution when injured with acidic material (17.7%) and should not wash the eye with an acidic solution when injured with alkaline material (19.9%, Table 2). Overall good knowledge was apparent among 426 participants (33.3%).

**Table 2:** Participants’ responses to knowledge items in the current study. * An asterisk indicates a correct response ¥ A multiple response item

Parameter	Category	N (%)
History of eye injury	Yes	305 (23.8%)
In which case should you go to the emergency room? ¥	When you have Persistent eye pain*	862 (67.4%)
	When you get Vision problem*	853 (66.7%)
	When you have Torn eyelid*	895 (70.0%)
	When you have mild pain	153 (12.0%)
	On eye movement not like the other*	777 (60.8%)
	Changes in the shape of the pupil*	606 (47.4%)
	Blood in the eye*	747 (58.4%)
	I will not go to the emergency	46 (3.6%)
	Presence of foreign body in the eye*	874 (68.3%)
	Others	15 (1.2%)
Which of the following is Symptoms of Scratched Eye? ¥	Eye pain*	770 (60.2%)
	Feeling like something stuck in your eye*	780 (61.0%)
	Blurred vision*	757 (59.2%)
	Darkness around the eye	163 (12.7%)
	Light sensitivity*	470 (36.7%)
	Do not know	190 (14.9%)
	Others	2 (0.2%)
If you get scratched in the eye, what is the first thing you will do?	I will rinse my eye with saline or clean water*	545 (42.6%)
	I will close my eye	350 (27.4%)
	I will Use anti-inflammatory eye drop	180 (14.1%)
	Clean the eye by cotton	60 (4.7%)
	Seek medical help (hospital or a doctor)	57 (4.5%)
	I will do no thing	86 (6.7%)
	Others	1 (0.1%)
In case of scratched Eye, you have to do more blink?	No	401 (31.4%)
	Yes*	319 (24.9%)
	Do not know	559 (43.7%)
In case of scratched eye, you have to rub your eye to help to remove any foreign objects?	No*	943 (73.7%)
	Yes	175 (13.7%)
	Do not know	161 (12.6%)
In case of scratched eye, you can wear your contact lens?	No*	1,115 (87.2%)
	Yes	47 (3.7%)
	Do not know	117 (9.1%)
In case of scratched eye, you can use redness-relieving eye drops?	No*	388 (30.3%)
	Yes	477 (37.3%)
	Do not know	414 (32.4%)
What should you do in case of blow to the eye?	Gently apply cold compress*	855 (66.8%)
	Put pressure on the eye	78 (6.1%)
	Gently apply warm compress	296 (23.1%)
	Seek medical help (hospital or a doctor)	35 (2.7%)
	Other	3 (0.2%)
	Do not know	12 (0.9%)
In case of cuts or puncture in the eye, apply a clean protective cover on your eye until you are able to see a doctor?	No	62 (4.8%)
	Yes*	1,007 (78.7%)
	Do not know	210 (16.4%)
You should remove anything stuck in the eye?	No*	613 (47.9%)
	Yes	279 (21.8%)
	Do not know	387 (30.3%)
In case of cuts or Puncture in the eye, which of the following is true?	Avoid rinsing the eye with water*	207 (16.2%)
	Put pressure on the eye protective covering	133 (10.4%)
	You have to take anti-inflammatory drugs	57 (4.5%)
	Nothing of the above is true	349 (27.3%)
	Do not know	533 (41.7%)
Chemical injury can cause ocular complications?	No	46 (3.6%)
	Yes*	980 (76.6%)
	Do not know	253 (19.8%)
What should be the first corrective action when a chemical injury occurs?	Wash with plenty of water*	539 (42.1%)
	Wash with little of water	36 (2.8%)
	Cover the eye	33 (2.6%)
	Go to emergency department	628 (49.1%)
	Use eye drops	37 (2.9%)
	Other	3 (0.2%)
	Do not know	3 (0.2%)
Alkaline injuries are more dangerous than acidic injuries?	No	189 (14.8%)
	Yes*	331 (25.9%)
	Do not know	759 (59.3%)
Locate and remove particles in the eye in case of chemical injury	No	301 (23.5%)
	Yes*	264 (20.6%)
	Do not know	714 (55.8%)
When injured with acidic material, wash with alkaline solution?	No*	227 (17.7%)
	Yes	210 (16.4%)
	Do not know	842 (65.8%)
When injured with alkaline material, wash with acid solution?	No*	254 (19.9%)
	Yes	168 (13.1%)
	Do not know	857 (67.0%)

Factors associated with knowledge

Results of the association analysis showed that good knowledge was significantly higher among females than males (36.5% vs. 27.2%, respectively, p < 0.001) and those without a history of eye injury than their peers with injuries (35.6% vs. 25.9%, respectively, p = 0.002). Additionally, good knowledge levels increased consistently with higher educational levels (15.6% among those with less than a secondary education, 24.6% among those with a secondary school education, 35.8% among university graduates, and 41.2% among post-graduates, p < 0.001). A good knowledge level was significantly higher among participants with the highest monthly income (42.3% for >15,000 SAR) compared to other categories of monthly incomes (30.6% for <5,000 SAR, 30.6% for 5,000 to 10,000 SAR and 33.5% for 10,000 to 15,000 SAR, p = 0.011), and single participants showed significantly higher knowledge levels (36.4%) than married (30.4%), divorced (26.8%) and widowed participants (18.2%, p = 0.042, Table 3).

**Table 3:** Factors associated with knowledge regarding eye injuries.

Parameter	Category	Poor, N = 896	Good, N = 443	p-value
Gender	Male	321 (72.8%)	120 (27.2%)	<0.001
	Female	532 (63.5%)	306 (36.5%)	
Age (y)	18 to 24	370 (65.6%)	194 (34.4%)	0.125
	25 to 29	101 (60.1%)	67 (39.9%)	
	30 to 34	89 (73.6%)	32 (26.4%)	
	35 to 45	151 (67.7%)	72 (32.3%)	
	> 45	142 (70.0%)	61 (30.0%)	
Marital status	Single	442 (63.6%)	253 (36.4%)	0.042
	Married	352 (69.6%)	154 (30.4%)	
	Divorced	41 (73.2%)	15 (26.8%)	
	Widow	18 (81.8%)	4 (18.2%)	
Educational level	< secondary	38 (84.4%)	7 (15.6%)	<0.001
	Secondary	193 (75.4%)	63 (24.6%)	
	University	555 (64.2%)	309 (35.8%)	
	Post-graduate	67 (58.8%)	47 (41.2%)	
Monthly income (SAR)	< 5,000	326 (69.4%)	144 (30.6%)	0.011
	5,000 to 10,000	229 (69.4%)	101 (30.6%)	
	10,000 to 15,000	163 (66.5%)	82 (33.5%)	
	> 15,000	135 (57.7%)	99 (42.3%)	
History of eye injury	No	627 (64.4%)	347 (35.6%)	0.002
	Yes	226 (74.1%)	79 (25.9%)	

On the multivariate analysis, good knowledge was predicted by the female gender (OR = 1.6, 95% CI, 1.3 to 2.1, p < 0.001), having a university degree (OR = 2.3, 95% CI, 1.1 to 5.8, p = 0.049) or a post-graduate degree (OR = 3.0, 95% CI, 1.3 to 8.1, p = 0.017), and having a monthly income of > 15,000 SAR (OR = 1.7, 95% CI, 1.2 to 2.4, p = 0.003). Contrastingly, participants with a history of eye injury were less likely to have good knowledge about eye injuries (OR = 0.6, 95% CI, 0.5 to 0.9, p = 0.003, Table 4).

**Table 4:** Predictors of good knowledge among participants in the current study.

Parameter	Category	OR	95% CI	p-value
Gender	Male	—	—	
	Female	1.62	1.25, 2.10	<0.001
Marital status	Married	—	—	
	Widow	0.59	0.16, 1.68	0.360
	Single	1.51	1.15, 1.98	0.003
	Divorced	0.88	0.45, 1.65	0.702
Educational level	< secondary	—	—	
	Secondary	1.39	0.61, 3.58	0.462
	University	2.31	1.06, 5.80	0.049
	Post-graduate	3.03	1.27, 8.09	0.017
Monthly income (SAR)	< 5,000	—	—	
	5,000 to 10,000	1.04	0.76, 1.43	0.797
	10,000 to 15,000	1.26	0.88, 1.81	0.203
	> 15,000	1.69	1.20, 2.39	0.003
History of eye injury	No	—	—	
	Yes	0.64	0.47, 0.85	0.003

## Discussion

This study aims to assess public knowledge and awareness regarding eye injury first aid and the significance of early management. Physical or chemical factors cause most eye injuries, and they are most frequently occupational [3,7]. Therefore, awareness about first aid can significantly reduce the devastating effects of eye injuries that could result in total blindness [8].

This study was conducted in three big cities Makkah, Jeddah, and Al-Taif. It shows that 23% of subjects have a history of eye injury, in contrast to other research done among the general population of Asser, which shows that a 15% of their participants have had a history of eye injuries [5]. Around three-quarters of our participants (73.7%) declined to rub their eyes to remove foreign objects. However, the contradictory results of the Asser study showed that 77.8% reported that they should rub their eye to remove any foreign object [5].

The main goal in managing penetrating eye injuries with Intra Ocular Foreign Body is always to preserve the globe and maintain ocular integrity. The next goal is to attain an excellent visual acuity outcome and to prevent further complications [9]. In case of cuts or penetrating eye injuries, three-quarters of participants said they would cover their eyes when they get cuts or punctures. In contrast to the Asser study, 87% answered correctly. Similar to the Asser study, in the case of cuts or penetrating eye injuries, nearly half of the participants advised against removing anything trapped in the punctured eye, but a few percent (16.2%) knew about avoiding washing the eyes with water [5].

Chemically induced ocular burn injuries can lead to complications such as neovascularization, inflammation, damage to the eyelid, cornea, conjunctiva, and corneal or stromal degeneration [10]. According to this study, over 76% of participants agreed that chemical eye damage could result in ocular complications, and in other studies from Asser 86.9% [5], from Jeddah 88.3% [11], and from Jazan 95.1% [12].

Regarding chemical ocular burn injuries, irrigating eyes with plenty of water and going to the emergency department were the most actions reported by the participants, 42% and 50%, respectively, similar to Asser's study, 46% and 47% [5]. While Jazan's study showed that the irrigation of the eye with a large amount of water was 60%, and going to the hospital was 28% [12]. In contrast, a study conducted in Jeddah showed that about 73% and 18% agreed that washing the eye with plenty of water is the first thing to do with chemical eye exposure and going to the emergency department, respectively [11].

Chemical ocular burns are usually caused by either acidic or alkaline agents [13,14]. Although both are devastating injuries, alkaline burns typically have a worse profile due to the tendency of alkaline chemicals to produce cellular membrane lysis, quickly penetrate the cornea and anterior chamber, and soften tissues [15,16]. Our study showed that 25% of the participant answered that alkaline eye injuries are much worse than acidic, while other studies showed 55% and 60% in Jeddah and Asser, respectively [5,11].

Regarding knowledge scores, respondents between 25 and 29-year-old showed good knowledge scores of approximately 40%, whereas Asser's survey revealed that the majority had approximately 32% knowledge scores [5]. However, the remaining respondents of all ages had a comparable percentage of good knowledge to the Asser study [5]. Results of the association analysis showed that good knowledge was significantly higher among higher educational levels. This finding agrees with a study conducted in Asser [5]. On the other hand, a good knowledge level was significantly higher among those without a history of eye injury than their peers with injuries. This contradictory finding of the Asser study showed a higher level of first aid awareness was substantially correlated with a history of eye injury [5].

In our study, good knowledge was statistically significant in the female gender (p < 0.001). However, most of the respondents were female in the current study, which may impact the results and is a limitation. In addition to single marital status (p < 0.003), university (p < 0.049), and postgraduate (p < 0.017) educational levels, monthly income of more than 15,000 SAR (p < 0.003) was the reported subgroup with higher total knowledge scores. On the other hand, populations with a history of eye injury were less likely to have good knowledge about eye injuries (p < 0.003), we think it might be a result of stressful events that affect their knowledge about first aid for an eye injury. Therefore, more research is needed to determine the relationship between these significant variables and their reliability.

Limitations

The current study has some limitations. First, only one region was surveyed; therefore, this might affect the generalizability of the study. However, our sample size and high response rate support the validity of these research findings. Second, the self-administered nature of the responses limited our study, the surroundings in which the questionnaire was completed was not closely monitored, and the possibility of discussing questions with other participants remained a reality.

## Conclusions

In conclusion, the study found public awareness of eye injury first aid was good in the western region. Also, the conception of going to a hospital or physician in the event of an eye injury is not prevalent among participants, which may be related to the reported physician's inadequate role in providing first aid knowledge. To increase the level of awareness among the populations toward first aid in eye injury, we encourage the use of multiple platform media to enhance awareness, make periodic educational campaigns and easy access to the ministry of health providing steps of first aid management.
